# Cardiotoxicity Associated with Immune Checkpoint Inhibitors

**DOI:** 10.3390/cancers15225487

**Published:** 2023-11-20

**Authors:** Shintaro Minegishi, Nobuyuki Horita, Tomoaki Ishigami, Kiyoshi Hibi

**Affiliations:** 1 Department of Cardiology, Yokohama City University Graduate School of Medicine, Yokohama 236-0004, Japan; tommmish@yokohama-cu.ac.jp (T.I.); hibikiyo@yokohama-cu.ac.jp (K.H.); 2 Chemotherapy Center, Yokohama City University Hospital, Yokohama 236-0004, Japan; horitano@yokohama-cu.ac.jp

Immune checkpoint inhibitors (ICIs) have shown significant efficacy in various cancers, including non-small cell lung cancer, small cell lung cancer, melanoma, classical Hodgkin lymphoma, head and neck squamous cell carcinoma, urothelial cancer, and renal cell carcinoma. Approved ICIs include monoclonal antibodies against programmed cell death-1 receptor (PD-1), its ligand (PD-L1), and cytotoxic T lymphocyte antigen-4 (CTLA-4). These antibodies have complementary mechanisms of action to one another, and so they are often used in combination. While ICIs have ushered in revolutionary advancements in cancer treatment, they can also cause several adverse effects known as immune-related adverse events (irAEs), with various organ manifestations. Among these, cardiotoxicity is a relatively infrequent adverse effect, occurring in less than 1% of cases, but it can pose serious concern.

While numerous studies have explored the cardiotoxicity associated with ICIs, and several review articles have been published, a systematic review conducted by Shalata et al. has demonstrated that the timing of cardiotoxicity onset varies depending on the specific type of cardiotoxicity, the treatment regimen, and the type of cancer under treatment [1]. One of the most severe cardiotoxicities linked to ICIs is myocarditis. Additional reported cardiotoxic effects encompass arrhythmias, conduction abnormalities, pericardial disease, and takotsubo cardiomyopathy. The median time until the presentation of ICI-related cardiotoxicity differs based on the treatment and cancer type, with all cancer types and treatments typically presenting within a median range of 1 to 31 weeks after initiating treatment [1]. Recent findings suggest a broad spectrum of timeframes for the onset of ICI-related cardiotoxicity, contingent upon the specific type of cardiotoxicity, the type of ICI, and the type of cancer being treated. While the majority of ICI-related cardiotoxicity cases emerge shortly after the initiation of treatment, a small subset of cases have been observed to develop after as late as 2 years [1].

Although the mechanism behind ICI-induced cardiotoxicity remains unresolved, it is likely to primarily involve CD4^+^-mediated T cell inflammation, with the most commonly produced cytokines including TNF-α, granzyme B, and IFN-γ. Additionally, experimental studies in mice suggest that PD-1 may play a role in preventing myocarditis, and PD-L1 expression in human myocardium could be implicated in protecting against immune-mediated myocardial injury and inflammation. The PD-1/PD-L1 pathway has been highlighted for its potential importance in preventing cardiac autoimmunity, though the CTLA-4 pathway is considered to be of lesser significance. Further advancement in basic research in this area is anticipated. There is ongoing research into identifying the risk factors and predictors of ICI-induced cardiotoxicity. A recent study suggested that interferon-γ and inflammasome-regulated proteins like GBP5 may have an important yet unrecognized role in the pathophysiology of myocarditis [2]. Laboratory tests often reveal elevated levels of BNP or NT-proBNP, signs of active inflammation such as elevated CRP and hepcidin, and increased troponin and CK-MB [1]. However, these markers lack specificity. While endomyocardial biopsy can be valuable, it is an invasive procedure and not routinely conducted. Cardiac magnetic resonance (CMR) is highly useful and considered the gold standard for imaging myocarditis. CMR can detect active inflammation characterized by increased capillary permeability, potential myocardial ischemia, and late gadolinium enhancement. In situations where CMR is unavailable, PET/CT serves as a viable alternative. The utilization of innovative approaches and techniques for investigating cardiovascular toxicity, such as employing human-induced pluripotent stem cell-derived cardiomyocytes and cardiac organoids in conjunction with conventional biomarker assessment and cardiovascular imaging, has the potential to enhance the safety and efficacy of cancer immunotherapies [3].

The increased use of ICIs has led to a rise in the incidence of irAEs. Real-world data on ICI therapy and cardiotoxicity in elderly cancer patients have been evaluated, indicating that ICI treatment is well tolerated in elderly patients, with cardiotoxicity rates comparable to those observed in the general population [4]. However, clinicians must remain vigilant not only to cardiotoxicity immediately after initiating therapy but also to suspicious symptoms several years after commencing ICI treatment. Effective management of ICI-related cardiotoxicity involves several considerations, including (1) identifying and assessing the type and severity of cardiotoxicity, (2) determining whether to withhold ICI therapy, (3) initiating steroid and immunosuppressive therapy, (4) commencing standard cardiac treatment, and (5) considering the possibility of restarting ICI therapy [5]. Concurrently, we are accumulating evidence on the significance of blood pressure control in cancer treatment from various perspectives, introducing the concept of onco-hypertension [6,7]. This concept encompasses monitoring blood pressure fluctuations in cancer patients, hypertension induced by anticancer therapy, and optimal blood pressure management in patients with concomitant hypertension and cancer. We have also validated the association between ICI use and hypertension and have demonstrated that it does not significantly increase risk in the short term [8]. In this study, we conducted a systematic literature search using various databases to evaluate the risk of hypertension associated with the initiation of ICI treatment in randomized controlled trials (RCTs). Hypertension severity was categorized according to specific criteria, and odds ratios for different grades of hypertension were analyzed through a meta-analysis. The study included 32 RCTs involving 19,810 cancer patients. The results indicated that, with a median follow-up of 36 months, the median overall survival for the ICI group w23018as 15 months. Importantly, the initiation of ICI treatment did not exhibit a significant association with the risk of hypertension, whether classified as grades I-V or grades III-V. However, it should be noted that cancer patients treated with ICIs face a heightened long-term risk of atherosclerosis and cardiometabolic diseases due to systemic inflammatory conditions and immune-related atheroma destabilization. Proprotein convertase subtilisin/kexin type 9 (PCSK9) plays a pivotal role in the atherosclerotic process and is also implicated in cancer progression and immune resistance [9]. As a novel pharmacological target for patients developing ICI-related atherosclerosis, PCSK9 inhibition therapy may offer potential benefits to these individuals.

Cancer remains one of the leading causes of death worldwide. Conversely, the prognosis and survival rates of cancer patients have significantly improved due to the development of cancer therapies, including molecular targeted drugs and immunotherapy. Consequently, the number of cancer survivors continues to increase each year. Cardiotoxicity associated with ICIs remains an ongoing area of study, with new information continually emerging. As a result, it is essential for both patients and physicians to stay well informed and for regular monitoring to be carried out during treatment. In cases where cardiac-related symptoms arise, addressing them promptly is of the utmost importance.
